# Operationalizing the ICF Core Sets for Autism and ADHD: A Multiple-Methods Feasibility Study

**DOI:** 10.1007/s10803-024-06717-4

**Published:** 2025-01-30

**Authors:** Lovisa Alehagen, John Hasslinger, Elina Wessman, Melissa Black, Karl Lundin Remnélius, Johan Helander, Eric Zander, Sven Bölte

**Affiliations:** 1 Center of Neurodevelopmental Disorders (KIND), Department of Women’s and Children’s Health, Centre for Psychiatry Research , Karolinska Institutet & Region Stockholm, Stockholm, Sweden; 2 Child and Adolescent Psychiatry, Stockholm Health Care Services, Region Stockholm, Stockholm, Sweden; 3 Curtin Autism Research Group, Curtin School of Allied Health, Curtin University, Perth, Australia

**Keywords:** Core sets, Functioning, International classification of functioning, disability and health, ICF, World health organization

## Abstract

The International Classification of Functioning, Disability and Health (ICF) is the World Health Organization’s (WHO) standard for assessing individual functioning. Over the last decade, the ICF has been made more accessible for autism and ADHD through the development and validation of tailored shorter ICF versions for these diagnoses, ICF Core Sets. To further enhance their applicability in research and practice, these Core Sets have been operationalized and implemented on an online platform, the ICF CoreSets platform. Here, we describe the iterative development process of the CoreSets platform. This multiple-methods study examines user feedback on the operationalization of the Core Sets and the feasibility of the CoreSets platform as a functional assessment for autism and ADHD. We collected a total of 678 assessments from the CoreSets platform. Individuals diagnosed with autism and/or ADHD, their relatives, participants from the general population, and professionals completed and provided feedback on the usability of the CoreSets platform. Qualitative feedback via interviews and focus groups were also collected. Qualitative data were analysed via content analysis, while quantitative data were examined using univariate and descriptive techniques. Findings show that the ICF CoreSets platform is feasible and user-friendly, but areas for improvement were also indicated, leading to additional refinement of the operationalization and platform. The operationalization of the ICF Core Sets and their implementation in the CoreSets platform appears adequate for use in research and practice, particularly after revisions indicated by future users, and is now ready for psychometric standardization.

**Supplementary Information:**

The online version contains supplementary material available at 10.1007/s10803-024-06717-4.

Neurodevelopmental conditions such as autism and attention-deficit hyperactivity disorder (ADHD) are common expressions of diverse maturation and function of the developing nervous system, being associated with challenges complying with mainstream societal demands and expectations (Bölte et al., 2023; Thomas et al., 2015; Zeidan et al., 2022). Individuals diagnosed with autism and ADHD exhibit variable individual functioning profiles which refer to how their activities (execution of tasks) and participation (involvement in life situations) unfold as a result of a complex interplay between their individual potentials as well as facilitators and barriers in the environment. Research demonstrates that clinical diagnosis is a too crude indicator of individual functioning and support needs in major life arenas, like education, employment, leisure, and family (Pellicano & den Houting, 2022). Thus, relying primarily on diagnoses when seeking to achieve sustainable social participation, mental and somatic health, and quality of life for autistic individuals and those with ADHD is short-sighted, as it does not account for the heterogeneity of the target group, the complexity of individual life situations, and differential environmental impacts.

Although evaluating an individual’s functioning is crucial for obtaining a personalized perspective, the concept of functioning has historically received little attention and continues to be regarded merely as an accessory to diagnosis. In addition, the concept of functioning is often confused with impairment or dysfunction, which conveys a negative perspective of the construct and fails to consider resource-oriented approaches or strategies to enhance participation. For instance, though assessment of functioning is included when considering a diagnosis of autism or ADHD, its primary purpose lies in informing decisions about the presence of qualitative impairment rather than examining individual functioning profiles which includes strengths, challenges and environmental influences. Thus, the recommended tools to support this decision-making in the core diagnostic manuals (i.e., DSM-5 and ICD-11), such as the World Health Organization Disability Assessment Schedule (WHODAS) (Üstün et al., 2010) and the ICF generic code set (Prodinger et al., 2016) have been developed with an impairment focus and are not tailored to specific diagnoses. Even other popular instruments with widespread use in research and practice that assess functioning often lack a holistic perspective on functioning. These commonly used assessments fail to consider environmental impact, potential individual strengths, or social participation (D’Arcy et al., 2022; Gleason & Coster, 2012), and are often generic in make-up, potentially being inadequate to capture diagnosis-specific factors that may influence functioning. These characteristics may limit their use for support planning and their acceptance by the target group (D’Arcy et al., 2022). In summary, for both diagnostic and intervention purposes in research and practice, as well as for a more individualized understanding of autism and ADHD, there is a need for an assessment of functioning using an internationally accepted standard that adopts a holistic approach, capturing essential facets of functioning relevant to the target populations.

More than two decades ago, the World Health Organization (WHO) developed the International Classification of Functioning, Disability and Health (ICF), a biopsychosocial framework of health-related functioning, aligning with a holistic and individualized view of a person’s potential and performance in light of their individual (body functions and body structures), personal (e.g., worldview, attitudes, age, gender), and external (social, attitudinal and physical) contexts (WHO, 2001, 2007). The ICF classification encompasses almost 1700 codes for body functions, body structures, activities and participation, and environmental factors enabling the generation of comprehensive individual functional profiles, comprising an individual’s strengths and challenges, and environmental facilitators and barriers (Bölte, 2023). The ICF has the authority of the WHO and enjoys the trust of all its 194 member states. It is also part of many national guidelines and legislation and bridges gaps between biomedical and neurodiversity views of neurodevelopmental conditions (Bölte et al., 2021; Leonardi et al., 2022). The ICF has influenced the development of policies and assessment frameworks for autism such as through the Federal German Participation Law (Bundesteilhabegesetz, 2016) and the Australian guidelines for autism assessment (AutismCRC, 2018). Additionally, the ICF is currently being evaluated in diagnostic and post-diagnostic services for autistic individuals in the United Kingdom (Autistica, 2021). Despite a growing body of research, the use of the ICF remains limited, which may be explained by several factors, primarily a lack of awareness among professionals, its comprehensive nature, and insufficient tailoring to specific diagnoses in its standard form. Efforts have been made to enhance the usability of the ICF, including the creation of ICF documentation tools (Selb Glässel & Escorpizo, 2015b), and the development of short lists of ICF codes specific to particular diagnoses. These so-called Core Sets improve diagnostic fit while at the same time reducing burden and complexity of use. Core Sets are developed using a standardized and rigorous procedure established by the WHO and the ICF Research Branch (Selb, Escorpizo, et al., 2015a), with many Core Sets now available for several conditions and contexts. Still, these initiatives do not operationalize ICF codes; thus challenges to implementation remain.

Initial comprehensive Core Sets for autism and ADHD were developed some years ago (Bölte et al., 2018, 2019). To support accessibility and to simplify application, a brief version was also developed, along with brief Core Sets tailored to specific age groups (preschool, 0–5 years; school-age, 6–16 years; older adolescent and adult, ≥ 17 years. Since their publication, the validity of the autism and ADHD Core Sets have been endorsed in several contexts, such as in employment and school environments (Black et al., 2019; Dreaver et al., 2020; Lee et al., 2020, 2023; Leifler et al., 2021; Scott et al., 2019) as a means to evaluate measures on functioning (D’Arcy et al., 2022; Hayden-Evans et al., 2022), to explore the effects of the COVID-19 pandemic on the autistic community (Fridell et al., 2022), and to investigate functional gender differences in autism (Lundin et al., 2021). The comprehensive and age-appropriate brief autism and ADHD Core Sets recently underwent their first revisions. The revised comprehensive Core Sets for autism and ADHD now contain 121 and 98 ICF codes respectively (Bölte et al., 2024a, 2024b). While these Core Sets are evidence-based standard selections of all ICF codes for assessing functioning in autism and ADHD, the utilization of the Core Sets remains constrained. To maximize usability and accessibility in research and practice for different groups of informants, ICF codes need to be operationalized into scorable items. Therefore, we operationalized the ICF Core Sets codes into items and implemented them as rating scales on a digital platform, the ICF CoreSets platform. In piloting the ICF CoreSets platform, we conducted both a quantitative validation study of the assessments and a multiple-methods study of user experiences and the feasibility of the platform. The quantitative validation is detailed elsewhere (Alehagen et al., 2025). Here, we focus on the development and initial usability and user experiences of the Core Sets operationalization and ICF CoreSets platform.

## Methods

### Design

**Fig. 1:**
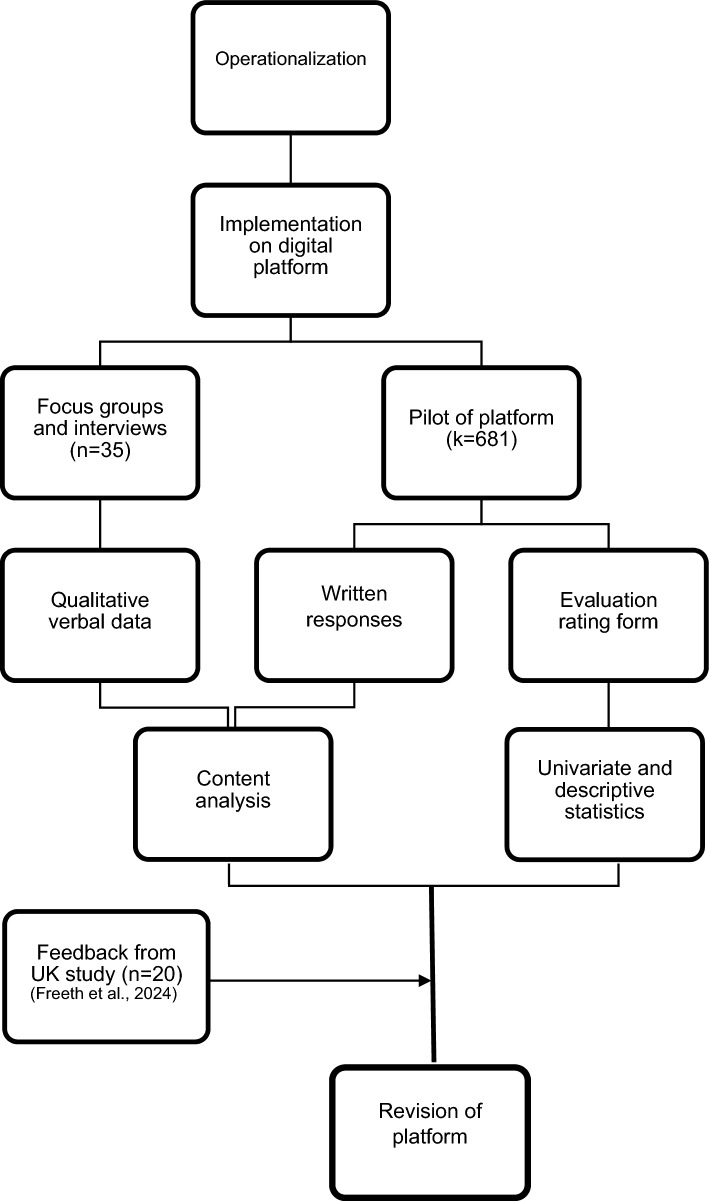
Flow-chart over the study process

In this multiple-methods feasibility study, we evaluated the ICF CoreSets platform for autism and ADHD. Following the operationalization of codes contained in the ICF Core Sets to scorable items and their implementation on the CoreSets platform, feedback on design, item clarity, flow, and overall user-friendliness was collected from Swedish autistic people, those with ADHD, professionals, other stakeholders, and the general population. Feasibility was also tested in autistic adults in the United Kingdom and is described elsewhere (Freeth et al., 2024). Data were collected via various sources, including (1) written feedback provided within the ICF CoreSets platform, (2) interviews and focus groups, and (3) a quantitative evaluation rating form. Based on the feedback received, both the platform and the Core Sets rating scales were revised to enhance usability and acceptability. The steps of the process are illustrated in Fig. 1.

Written informed consent was collected from all participants and/or their parents or legal guardians prior to participation. Ethical approval for the study was obtained from the Swedish Ethical Review Authority (2021-03998 ), and the study was pre-registered on the Open Science Framework (10.17605/OSF.IO/TNDZ2).

## CoreSets Platform Development

### Operationalization of Core Sets

The ICF Core Set codes for autism and ADHD were operationalized into a set of items for both self- and informant report. Given the absence of universal guidelines for operationalizing ICF codes, an iterative process involving stakeholders and professionals from different countries was undertaken, using common questionnaire design principles (Krosnick & Presser, 2010). First, two authors (SB, EZ) transformed the codes into assessable items guided by the principle of generating as few items as possible but also as many as needed to adequately and closely reflect the relevant construct described in the ICF manual. The authors sought to focus on one item in each question, use simple language, keep the questions brief, and create a shared introduction to sections of questions. If the 2nd level ICF codes in the Core Sets included 3rd and 4th level coding details, these were consulted and transformed into questions to maximize the content validity of the generated items. Some codes yielded a single item (e.g., d130 “Copying” [Imitating or mimicking]), while others were broader and more complex, resulting in multiple questions (e.g., d350 “Conversation” with 7 questions). Insights from the development of the autism and ADHD Core Sets (Bölte et al., 2018, 2019) also informed this operationalization process. Subsequently, an open and iterative process followed, involving an international group of professionals experienced with both ICF and neurodevelopmental conditions from Australia, Germany and Sweden, as well as stakeholders and clinicians from Sweden. Their feedback and opinions were considered, resulting in a revision of the items. A total of 123 codes from the autism and ADHD Core Sets were operationalized into 286 items. Operationalization of the age-specific Core Sets resulted in between 141 (ADHD, age 0 to 5) and 233 items (autism and ADHD combined, age > 16,) which were derived from 62 to 89 ICF codes.

For quantification of functioning, the ICF recommends qualifiers (WHO, 2001) in the form of five-point scales to rate functional abilities and disabilities in an individual’s daily life. However, these qualifiers have limitations in assessing overall functioning, as they primarily measure impairment and do not adequately capture strengths. In addition, standard ICF qualifiers lack both space for broader quantification of functioning and elasticity to assess functional change. To address this, the ICF CoreSets platform employs an adapted numeric rating scale (McCaffery & Beebe, 1989), whereby an 11-point Likert Scale is used, assessing both hindering factors/challenges and facilitating factors/strengths. Participants scored items on an ordinal scale from 0 to 10 for body functions and activities and participation, with 0 to 5 indicating challenges, 6 to 8 indicating normative functioning, and 9 to10 indicating that the factor represents a strength. Environmental factors were scored from − 5 to + 5 (increasingly hindering: −1 to-5, increasingly facilitating: 1 to 5, “0” for neutral). The body function sensory processing was also scored from − 5 to + 5 and unusual movements from 0 to −5.

### Implementation on Platform

A cloud-based assessment platform, the ICF CoreSets platform, was developed. The platform integrates the operationalized ICF Core Sets in the form of scorable items as online rating scales. The platform was designed to be self-explanatory, with no need for informant training or instructions given by a professional. On the platform, there are 15 functional assessment versions corresponding to the respondent type (self or proxy), age range, and diagnosis. These include five versions for autism and ADHD, each comprising age-appropriate self-reports (6–16 years and adult) and proxy-rating versions (0–5 years, 6–16 years, and adult). There are also five additional autism and ADHD combined scales (6–16 years and adult as self-reports and 0–5 years, 6–16 years and adult as proxy report). Participants gain access to an assessment upon receiving a link to the platform by invitation of a professional. In addition to Likert-scale ratings, each item includes a comment box for participant feedback or clarification of replies. Participants can complete the entire assessment at one point in time or save their progress and continue at a later time of their choice. Before the revisions, the assessment included a single open-ended question positioned at the end, where the participant could provide any additional functional information of importance from their perspective.

## User Experience and Feasibility Evaluation

### Participants

Individuals diagnosed with autism and/or ADHD (self-report), relatives of autistic individuals and/or those with ADHD (proxy-report), and individuals from the general population (self or proxy-report) were eligible to participate in the study. Autistic individuals, individuals with ADHD, and their caregivers/guardians were recruited through cooperation with interest organisations, a clinical unit attached to the research centre, and via collaborations with service providers such as child and adolescent and adult psychiatry. Individuals from the general population were recruited in cooperation with the data collection and marketing company PFM Research, who selects individuals from nationally representative panels. Inclusion criteria for individuals diagnosed with autism and/or ADHD was a community diagnosis of autism, ADHD, or both, or a self-identified diagnosis of autism and/or ADHD. Relatives were required to be a caregiver or guardian of an individual with a community diagnosis of autism and/or ADHD. There were no exclusion criteria for the general population group, except for the self-reported presence of an autism or ADHD diagnosis. Participants could undergo assessment by multiple relatives, and they could also conduct more than one assessment. For instance, a participant might complete both a self-report and a proxy-report for a relative. In total, 678 assessments were collected, including 311 assessments of individuals with autism and/or ADHD and 367 assessments of neurotypical individuals, see Table 1.

**Table 1:** Overview of raters’ sociodemographic data per ICF core set versions

Core Set – age-version		Self-rating		Proxy-rating		
	Ratings	Female: male (other^a^)	Age mean (sd)	Female: male (other^a^)	Age mean (sd)	Missing data^b^
ADHD – 6–16 years (156 items)	k = 17	1:3	12.00 (2.58)	6:0	42.17 (1.47)	7
ADHD – adult (170 items)	k = 49	18:3 (3)	37.37 (12.78)	13:7	46.05 (18.03)	5
ASD – 0–5 years (185 items)	k = 2	–	–	1:1	40.50 (9.19)	0
ASD – 6–16 years (202 items)	k = 19	2:4 (1)	14.00 (1.53)	8:3	44.27 (10.01)	1
ASD - adult (218 items)	k = 78	34:8 (3)	39.20 (12.33)	15:11	47.19 (18.58)	7
ASD + ADHD – 6–16 years (213 items)	k = 31	1:3	13.50 (2.65)	14:6	46.80 (11.88)	7
ASD + ADHD - adult (232 items)	k = 115	47:13 (3)	37.68 (12.91)	22:10	47.56 (14.31)	20
ASD + ADHD – common^c^ (170 items)	k = 367	110:101 (1)	43.31 (15.79)	79:72 (4^a^)	45.10 (15.27)	0

No Core Sets for the ADHD – 0–5 years, nor the ASD + ADHD – 0–5 version were completed

^a^Includes responses: non-binary, other, unsure and don’t want to answer

^b^Missing data from Sociodemographic survey

^c^Used for the general population

Individuals who completed the ICF Core Sets platform were also invited to engage in an interview or focus group about their user experience of the platform. A group of participants who had consented to focus group participation were contacted. A total of 35 participants engaged in the interviews/focus groups, including 12 neurodivergent people (2: male, 9: female, 1: non-binary) and 11 relatives who were partners, friends, and parents of neurodivergent individuals. For privacy considerations, only data pertaining to gender and the relative’s relationship to the assessed individual were gathered. Additionally, a subgroup of professionals (psychologists and special education teachers, (*n* = 11) was recruited to participate in individual interviews or focus groups. The professionals only engaged with the platform for evaluation purposes, hence, their assessment responses were not included in the overall count of 678 assessments. They were recruited via professional networks.

### Data Collection Materials

Online Comments: Comments made regarding specific items or the whole platform during the assessment via comment boxes within the ICF CoreSets platform were collected from all responders (diagnosed participants, their relatives, and general population) if provided by participants. This participant feedback was reviewed for any opinions or comments concerning the construction and contents of the rating scales.

Focus Groups and Individual Interviews: A combination of focus groups and interviews were conducted in order to enrich the data and broaden understanding (Lambert & Loiselle, 2008). Between five and 10 participants were invited per group (Kreuger, 1994; Morgan, 1997), however, in instances where smaller groups of two participants occurred, the sessions were conducted as planned. Focus groups and individual interviews were conducted online via Zoom. The video feature was recommended but participants could choose not to use it. The first author moderated the meetings while a research assistant (JHe) took notes. Focus groups and interviews followed a semi-structured format, allowing for flexibility in question order while maintaining consistent overall content. The primary focus was on evaluating the wording and content of the rating scales, as well as the design of the ICF CoreSets platform. Sample questions included: “How did you find the wording of the questions?”, “Is there anything related to your daily functioning that was not covered in the form?”, “How well did you understand the questionnaire?”, “What would facilitate understanding?”. Phrases such as “Explain more” or “How do you mean?” were used to clarify comments and deepen understanding.

Standard Evaluation Form: A standardised evaluation form, developed by the research team for the purposes of the study and inspired by the System Usability Scale (Brooke, 1996) captured information concerning the operationalization and platform’s feasibility for: (i) time spent to complete assessment, (ii) navigation, (iii) familiarity, (iv) consistency, (v) visual clarity, (vi) flexibility and efficiency, (vii) overall feasibility. Items were scored on a scale from 1 to 4, with higher scores indicating a more user-friendly experience. Hence, a mean score above 2,5 reflects an increasingly positive opinion, whereas a mean score of 2,5 and below reflects an increasingly critical view.

### Procedure

Prior to participating individuals received study information and were required to provide written informed consent (or assent with guardian consent). Subsequently, participants received a link to the ICF CoreSets platform. Within the ICF CoreSets platform, participants completed the most appropriate version of the rating scale in accordance with their respondent type. The general population group responded to a version containing 170 items (brief autism/ADHD combined version), while the number of items for neurodivergent group and their relatives/guardians, varied between 156 and 232, depending on age and diagnosis, and thus corresponding ICF CoreSet operationalization. Participants were also sent a sociodemographic form and the evaluation rating form. Following the completion of the ICF CoreSets platform, individuals diagnosed with autism and/or ADHD and relatives were invited to participate in an interview or focus groups after completing the ICF assessment. For those who could not attend focus groups or preferred other formats, individual interviews were offered. A select number of professionals were also invited to view the platform and provide feedback via an interview or focus group. Eight focus groups and five interviews were conducted, with group sizes ranging between 2 and 7 participants.

### Data Analysis

#### Qualitative Data

Qualitative data comprised both written text collected from the ICF CoreSets platform and verbal data from interviews/focus groups. Written responses from the ICF CoreSets platform were exported for analysis. First, text within the text boxes of the rating scales was analysed. Sub-domains of the assessment were grouped, and comments related to each item were reviewed. The focus was on opinions specific to the items, excluding any personal matters. Furthermore, the last text box in the rating scale was analysed. Meaning units were identified, condensed, and coded until categories and sub-categories were developed. The findings were discussed among the authors (LA, JHa and SB), which laid the basis for revisions on the ICF CoreSets platform. Finally, qualitative data generated from the feedback derived from the UK study (Freeth et al., 2024), which exhibited significant overlap with the feedback from Sweden, was evaluated and added to the revision process.

Interviews and focus groups were transcribed verbatim by the first author (2 interviews) and by an external transcriber (11 interviews). Transcripts were cross-checked against the original recordings to ensure accuracy. The focus groups and interviews were analyzed using qualitative content analysis (Graneheim & Lundman, 2004; Elo & Kyngäs, 2008) with a primary focus on the manifest content level of the text (Given, 2008). Initially, two authors (LA, JHa), read each transcript and then re-read them and identified meaning units that were condensed and coded. The codes were then sorted into categories and sub-categories. The analysis of all following transcripts utilized categories and sub-categories that had been established during the analyses of previous transcripts as well as establishing new ones. When new categories emerged, meaning units from previous transcripts were reassessed in the light of the new categories. Hence, the analysis process combined both an inductive and a deductive approach. To ensure rigor, findings from each author (LA, JHa) were compared across sources (neurodivergent, relative, professional) before all results were consolidated. In case of disagreement, the categories, sub-categories, and codes were discussed until consensus was reached.

#### Quantitative Data

Data collected via the standardized evaluation form were analyzed quantitatively. Six questions addressing various aspects of user-friendliness on the standardized evaluation form were examined using a descriptive univariate analysis. Ratings from the different diagnostic and age-group versions of the ICF Core Sets were consolidated into a single group, regardless of the number of items. Mean scores were then compared to those of the general population group, with separate analyses for self-ratings and proxy ratings.

## Results

### Qualitative Results

Content analysis yielded 4 categories and 11 sub-categories. A summary of the categories along with illustrative quotes are presented in Supplementary material (table S1). To support the categories, participant type (N = neurodivergent, R = relative, or P = professional) was used as an identifier for each quote.

### Usability

*The platform*: Many participants expressed a positive view of the platform, citing easy navigation, clear instructions, and visual appeal. As illustrated by two participants: “The text is clear, and… I think so too, especially with the fonts and such. So, I thought it was good.” (N8) and “It’s very neat and it’s very clear what to do.” (P9). The importance of considering diverse behaviours within diagnostic categories was emphazised. The majority of professionals believed the ICF CoreSets platform could aid in assessments and facilitate a shift away from a purely diagnostic approach toward a more individualized perspective. One professional expressed: “I believe there’s great value in… if we can just get this out on a broader scale, we can move away from the notion that an assessment is solely about assigning a diagnosis. What’s stated in the assessment is a diagnosis, but then that’s something very general. We want the help to move forward.” (P8). Discussions highlighted the potential of the platform as a tool for interprofessional communication and overall, the work was deemed relevant by all groups. “I think it’s incredibly relevant, and I’m really happy that you’re working on this. I’m very excited to see what the results will be.” (R11).

*Feedback*: A prevailing opinion was the desire to receive a summary of the answers and some form of assessment results. Some participants indicated a desire for a review, as they had forgotten their answers or wanted to reflect on them, as one participant stated: “Something I missed a little was perhaps that you could go back to your answers so you could reflect on it.” (N5). Others sought to show or compare their responses with others, like this relative said: “One doesn’t receive a summary of one’s responses; it would be interesting to compare my answers with my children’s.” (R9).

*Enhanced accessibility*: Participants emphasized improving platform accessibility to enable a broader range of individuals to participate. Recommendations included allowing users to customize colors for better clarity in the response scale and implementing a screen reader to enhance accessibility for those with visual impairments. One participant expressed: “I think about people who don’t have complete color vision. Isn’t it impossible for many people to distinguish between red and green?“ (R5). A dialogue about this arose between two other focus group participants:“I was wondering, was there support for resolution or audio description for those who…?”, “Yes, I think that would have been helpful. Especially if it read out the questions, for example.”, “Because that excludes quite a few people from participating, given that it doesn’t exist.” (N12 and N10).

### Items

*Appreciated items*: Several positive opinions emerged regarding the items. Participants appreciated the comprehensive coverage across various domains and the inclusion of strengths alongside challenges. The incorporation of environmental factors was mentioned as a positive aspect by all groups, given their significant impact on overall functioning. For example, one neurodivergent participant said: “I think it was good that questions were asked about how one is affected by different aspects of society.” (N3), while a relative expressed: “I would say, it’s a really crucial area… because the environment makes all the difference, and context and surroundings are where difficulties arise.” (R8). Also the professionals lifted this as an important area: ”Recognizing that the disability arises in relation to the environment is crucial, and it should be considered as something that needs attention.” (P8). Other items that were appreciated were those for social participation, sleep and perception, as described by this participant: “You have quite a few questions related to participation, self-determination, and the ability to influence society. I think those aspects are very important because they’re not always thoroughly considered in typical assessments.” (P10).

*Length*: Although opinions varied, many participants expressed that the rating scales were overly comprehensive, illustrated by these two quotes: “…she [the daughter] thought there were a lot of rephrasing, like asking about the same thing with different sentences and from different angles…it became too cumbersome and repetitive. But as I said, that was her perspective… I didn’t react to it because I love forms.” (R11) and “It also depends on how sensitive one is to answering these kinds of questions. I think perhaps people who are supposed to answer this might be more okay with it than I am; I just get tired of these things and feel like enough is enough.” (P9).

Recommendations for condensing the rating scales included creating different assessment versions based on expected levels of functioning or more clearly categorizing questions suitable for different age groups. Consolidating related topics into single questions and removing unnecessary ones were also suggested.

*Requested additional items*: Participants identified several topics that were either absent or deserving more emphasis. Two recurring themes emerged: masking and energy regulation. Many participants believed that these themes significantly influenced their functioning and should be integrated into the assessment process. Some participants expressed that masking - adopting behaviours to appear more neurotypical in the presence of neurotypical individuals - and managing volatile energy levels had a substantial impact on their responses to assessment items. Consequently, answers could vary significantly based on whether an individual was masking and the level of energy they experienced on a given day or moment. These themes were correlated for many participants, highlighting how masking behavior can lead to a more rapid depletion of energy. Like one participant said: “It doesn’t address things that aren’t very visible externally, such as masking and the fatigue that can occur during the overload…” (R10).

*Neurotypical perspective*: Some participants raised concerns about potential bias towards favouring a neurotypical perspective. For example, valuing a more socially active lifestyle than personally desired. For example, one participant said: “Is it really better to be more outgoing and social? I might not want to be super social all the time, but I don’t see it as a problem.” (N8). Specific traits, such as sociability and extraversion, were rated as strengths, while stereotypic movements (e.g., hand-wringing, flapping, finger-waving, rocking, stimming), were rated as being associated with low functioning, which some perceived as indicative of this bias. One participant expressed: “…stimming is perceived as something negative…even though it is natural and healthy.” (N9). Some critiqued the rating scales’ underlying design, which involves individuals comparing themselves to others on various items. Like one participant said: “If others engage in doing those things, it’s quite irrelevant to me…” (N7). They contended that this approach lacks neurodiversity-friendliness. Instead of conforming to societal expectations of functionality, they advocated for exploring one’s preferred lifestyle and values, emphasizing that others’ behaviours or values should not necessarily be relevant.

### To Respond

*Response challenges*: Numerous participants highlighted difficulties in rating their functioning at all, owing to its variable nature, and mentioned factors such as situational context, motivation, and mood. Consequently, assigning a single numerical score to each item may be misleading, as functioning levels can fluctuate significantly from day to day. Participants expressed: “It’s one answer when I have energy and feel fully… well and all that, but it’s a completely different answer if I’m overstimulated or exhausted.“(N7), and: “It varies depending on where he is. Whether he’s with me or someone outside the home, it’s like dealing with two completely different people.” (R1). Additionally, some respondents found the items overly broad or general in their formulation, leading to challenges in providing accurate answers. Specific issues included unclear wording, and instances of compound questions (e.g., “I am X and Y”). One participant said: “I also thought that certain questions with double assertions were challenging to evaluate. They weren’t quite the same thing, which made them difficult to answer.” (R9). Some participants also experienced ambiguity regarding the timeframe (e.g., questions related to school functioning when the respondent was not currently attending school), like this participant said: “It was formulated in such a way that it was difficult to know whether one should answer based on how it was when they were in school or if it only applied to those currently attending school. So, it was a bit difficult to answer, you know.” (N12).

*Item scaling*: The response scale elicited both positive and negative opinions. A majority of respondents found the use of colors helpful, as these participants described: “I thought it was good that it was divided like this in colors because it made it a bit clearer to overview.” (N12), and “So, I think it’s pedagogical to use different colors. And it also becomes clear what falls within the normal range, which gives you an idea of how the scale is graded.” (R7). Moreover, most participants appreciated the comment field alongside each item, allowing them to provide clarifications when necessary. However, some users found the comment field too small for expressing their thoughts fully, like this participant said: “I wrote a lot of comments, but the characters didn’t always suffice for me. However, I understand that you don’t want essays for every little question.” (R5). The omit buttons beneath the response scale (‘Not applicable’ and ‘Unknown’) raised questions, as many individuals did not understand their intended functions and found the wording difficult to understand: “What is the difference between ‘unknown’ and ‘not applicable’?” (N8). It was recommended to either simplify the wording or to provide explicit instructions on their usage. Opinions on the steps in the response scale were mixed. Some participants found the scale too detailed, making it hard to distinguish differences between steps. One participant said: “There are so many steps, how are you supposed to determine whether it’s a one, two, or three; perhaps there are too many steps involved.” (R10). Others deemed the 11-point scale appropriate. Furthermore, concerns arose about the scale’s asymmetry, with more space allocated to challenges than strengths. The words underneath the scale “Atypical-Typical-High function” were confusing too many. Some also found them marginalising and judgemental.

### Instructions

*Clarity*: Participants generally found the instructions clear and the rating scale description adequate, like this participant: “I thought the instructions were good. I understood what I was supposed to do.” (N3). Suggestions for improvement included providing guidance on how to approach the assessment questions, adding a feature for requesting further explanations, and giving more details on the rating scales’ length and content. One participant expressed: “For my part, it would be good to have a bit more overview of the test layout before I started. Something like a button that says, ‘Look here to see what type of questions are coming.” (R7).

*Progress bar*: The progress bar was well-liked and found to be helpful by some participants: “It was like a progress bar at the top, and that was comforting.” (N5). However, some suggested enhancement for the progress bar, like showing the remaining number of items instead of pages.

### Quantitative Results

In both neurodivergent and general population groups, including both self- and proxy ratings, the mean scores generally indicate an acceptable view of the platform. Items related to design (items 2–5) were consistently rated highly across all groups, with mean scores ranging from 2.9 to 3.41 (max score of 4), indicating a favorable view of the platform’s design. Item 1 (“What do you think about the time it took to answer the questionnaire?”) received the lowest scores across all groups, suggesting that respondents found the rating process somewhat extensive with mean scores from 2.32 to 2.59. Approximately 60% of the neurodivergent groups, both self-and proxy-rated assigned this item with a score of 1 or 2. The summarizing item 6 (“In summary, how user-friendly was the ICF CoreSets tool?”) was scored between 2.94 and 3.26 and around 80–90% of all participants rated the overall user-friendliness as either 3 or 4. For more information, see Table 2.

**Table 2:** Evaluation scores self and proxy ratings, neurodivergent^a^ vs. general population^b^

	Self-rating neurodivergent^c^ k = 145					Proxy-rating neurodivergent^c^ k = 103				
	Mean (sd)	Frequency (%) per response				Mean (sd)	Frequency (%) per response			
		1	2	3	4		1	2	3	4
1. What do you think about the time it took to answer the questionnaire?	2.36 (0.82)	19 (13.1)	68 (46.9)	45 (31)	13 (9)	2.32 (0.69)	9 (8.7)	56 (54.4)	34 (33)	4 (3.9)
2. How easy was it to navigate around the questionnaire?	2.96 (0.74)	5 (3.4)	28 (19.3)	80 (55.2)	32 (22.1)	2.93 (0.73)	3 (2.9)	22 (21.4)	57 (55.3)	21 (20.4)
3. How intuitive was the ICF CoreSets tool for you?	2.90 (0.75)	8 (5.5)	24 (16.6)	87 (60)	26 (17.9)	2.97 (0.68)	2 (1.9)	19 (18.4)	62 (60.2)	20 (19.4)
4. How consistent in its design was the ICF CoreSets tool?	2.99 (0.68)	5 (3.4)	19 (13.1)	93 (64.1)	28 (19.3)	3.12 (0.60)	1 (1)	10 (9.7)	68 (66)	24 (23.3)
5. How easy was it to orientate on the page?	3.22 (0.72)	4 (2.8)	13 (9)	75 (51.7)	53 (36.6)	3.39 (0.60)	0 (0)	6 (5.8)	51 (49.5)	46 (44.7)
6. How user-friendly was the ICF CoreSets tool?	2.94 (0.70)	5 (3.4)	24 (16.6)	90 (62.1)	26 (17.9)	3.17 (0.61)	1 (1)	9 (8.7)	65 (63.1)	28 (27.2)

	Self-rating general population^d^ k = 195					Proxy-rating general population^d^ k = 130				
1. What do you think about the time it took to answer the questionnaire?	2.59 (0.82)	16 (8.2)	72 (36.9)	82 (42.1)	25 (12.8)	2.48 (0.77)	11 (8.5)	57 (43.8)	51 (39.2)	11 (8.5)
2. How easy was it to navigate around the questionnaire?	3.33 (0.70)	3 (1.5)	17 (8.7)	88 (45.1)	87 (44.6)	3.09 (0.74)	4 (3.1)	18 (13.8)	70 (53.8)	38 (29.2)
3. How intuitive was the ICF CoreSets tool for you?	3.09 (0.64)	3 (1.5)	23 (11.8)	123 (63.1)	46 (23.6)	3.02 (0.72)	4 (3.1)	20 (15.4)	76 (58.5)	30 (23.1)
4. How consistent in its design was the ICF CoreSets tool?	3.14 (0.66)	2 (1)	24 (12.3)	114 (58.5)	55 (28.2)	3.03 (0.75)	8 (6.2)	10 (7.7)	82 (63.1)	30 (23.1)
5. How easy was it to orientate on the page?	3.41 (0.63)	1 (0.5)	12 (6.2)	88 (45.1)	94 (48.2)	3.27 (0.69)	2 (1.5)	12 (9.2)	65 (50)	51 (39.2)
6. How user-friendly was the ICF CoreSets tool?	3.26 (0.63)	2 (1)	14 (7.2)	110 (56.4)	69 (35.4)	3.14 (0.73)	4 (3.1)	15 (11.5)	70 (53.8)	41 (31.5)

All items were rated on a scale 1–4. Higher scores indicated a more positive/satisfactory experience. Mean scores below 2.5 indicate a negative opinion (e.g., too long, not user-friendly) while scores above 2.5 indicate a positive opinion (e.g., good length, user-friendly) ,

^a^k = 59 were excluded due to missing data and k = 4 due to the absence of a reported neurodivergent diagnosis

^b^k = 42 were excluded due to the reporting of a neurodivergent diagnosis

^c^For the neurodivergent group the number of items varied between 156**–**232 (average 211), depending on age and diagnosis version

^d^The version for the general population group always consisted of 170 items

### Revisions

Following data analysis, decisions were made regarding the implementation of suggested revisions to improve the ICF CoreSets platform. These decisions were deliberated by three authors (LA, JHa, SB) based on the following criteria: (a) Alignment of suggestions with the original ICF code and overarching ICF philosophy, (b) Frequency of reiterated suggestions, (c) Practical feasibility of incorporating suggestions into the rating scales (including collaboration with IT technicians to assess proposed design enhancements). Subsequently, all suggestions underwent scrutiny by the authors (LA, JHa, SB) who made the final decisions regarding the revisions. Moreover, a language custodian reviewed the instructions for the ICF Core Sets rating scales, focusing on enhancing language accessibility. The recommendations were collectively evaluated by the authors (LA, JHa, SB) to determine which should be integrated into the final version of the ICF CoreSets platform. Lastly, a test trial took place at the research centre, involving professionals who were asked to complete the assessment and provide feedback on the revised version. This process resulted in additional minor adjustments.

### ICF Core Set Operationalization to Rating Scales

Findings led to several revisions to the rating scales. For example, in response to feedback regarding a neurotypical perspective on behaviors such as stereotypic or repetitive movements (e.g., stimming), the response scale for that item was modified. As a result, the presence of such behaviors can now be rated as both hindering and helping. While the comment field for each item remained unchanged, additional free-text boxes were introduced after each domain, to allow for more additional information. After each section of items for ICF domains for body functions, activities and participation, and environmental factors, two open-ended questions, allowing participants to provide additional personal information related to the domain and describe their primary support needs in that area, were added. The final open-ended question in the assessment was retained, allowing participants to provide any additional functional information they considered important from their perspective. In total, 54 items were reworded, 17 were removed and 14 were added (for example regarding masking and energy regulation). See Table 3 and Supplementary material (table S2-S4) for more information.

**Table 3:** Changes to items, per type and domain

Type of change	Body function	Activity & participation	Environmental factors	Total
Clarified examples	3	11	1	15
Rephrasing	13	17	6	36
Rewording	23	29	2	54
New items	2	9	3	14
Removed items	1	15	1	17
Other	2^a^	1^b^	4^c^	7
Total	44	82	17	143

Rephrasing entails more comprehensive changes to the item, while Rewording entails simple changes such as editing single words. Clarified examples entails changes or additions to examples given

^a^sub-domain renamed and change of response scale in on sub-domain

^b^addendum to header

^c^preamble added

### Platform

Several revisions were also made to the platform following participant feedback. For instance, an accessibility tool was implemented with possibility to change for example contrast, text spacing, add a reading mask and a screen reader. The colors of the response scale were clarified and the words underneath the scale (Atypical- Typical – High functioning) were removed. Instructions and items were clarified to better match the requests, for example the time frame of the assessment. The wording of the omit buttons was changed from “Unknown” and “Not applicable” to “I don’t know” and “Not relevant” and instructions on how to use them were added. Further, a completely new feature, a result summary, was added. An algorithm was created to extract information about reported strengths, challenges, hindering factors, and helping factors. Additionally, the summary incorporates the text from the free text box at the end of each domain, specifically focusing on primary support needs. The result summary gives an overview of the ICF assessment, applicable to both self-assessed individuals and those assessing a relative. For further details on implemented revisions, please refer to Supplementary material (table S5).

### Feedback not Implemented

Some suggestions could not be implemented due to various factors. These considerations included practical limitations, incompatibility with other suggestions, and alignment issues with the existing ICF Core Sets. For instance, incorporating new areas not covered by the Core Sets was not viable. Consequently, requests to include items related to, for example, school settings, mental health, and coping strategies were not integrated. Additionally, it was not possible to remove themes or codes from the scale to shorten it, as these codes were determined through a rigorous global process and fall outside the scope of this feasibility study. Therefore, the rating scale remained comprehensive. However, instructions regarding the option to pause the assessment were clarified, and automatic reminder emails were implemented to encourage participants to complete the assessment later. Moreover, the response scale remained unchanged for most items, with greater emphasis on challenges and hindering factors than on strengths and helping factors. The number of scale steps was also retained.

## Discussion

Traditionally, neurodevelopmental conditions have been predominantly conceptualized as categorical entities applying a medical model. However, research and lived experience accounts highlight that clinical diagnosis alone is too simplistic to capture phenotypic heterogeneity, exemplifying why using a biopsychosocial model of individual functioning, particularly as a basis to plan support measures for achieving participation is meaningful. Currently, endorsed by the WHO and its 194 member states, the ICF serves as the global standard for health-related functioning assessment. However, there are multiple barriers to implementation of the ICF, such as limited awareness among professionals, but also its complexity and lack of diagnoses link. Thus, for over a decade, our research centre in collaboration with WHO, the ICF Research Branch, and researchers, clinicians and stakeholders around the world have worked to enhance the accessibility of the ICF for autism and ADHD (Bölte et al., 2024a, 2024b; Bölte et al., 2018; Bölte et al., 2019). This effort involved developing and validating Core Sets tailored for these diagnoses. In order to further enhance applicability of the ICF in autism and ADHD, we continued our efforts by operationalizing the codes of these Core Sets and implemented them on a platform as digital rating scales. In this multiple-methods study, we evaluated the operationalization and implementation for feasibility in terms of clarity, flow, and user-friendliness. Feedback from study participants (neurodivergent, relatives, professionals, general population) informed revisions of the items and the platform.

Consensus from both qualitative and quantitative data indicates that the ICF CoreSets platform and its aims were generally received positively. Qualitative data indicated that participants appreciated the comprehensive coverage of functioning domains and its ability to capture strengths and environmental impacts on functioning, while quantitative data indicates that it is generally acceptable and user-friendly. A recurring theme revolved around the diagnostic approach, with neurodivergent individuals and their relatives emphasizing the importance of recognizing the uniqueness of individuals irrespective of diagnosis. Professionals concurred and expressed that relying solely on diagnostic information was insufficient for designing effective interventions, reinforcing a need for more individualized functional assessment. These findings align with research that challenges an exclusively diagnostic perspective, highlights the importance of the neurodiversity perspective in the context of neurodevelopmental conditions (Bölte et al., 2021), and reaffirms the potential of the functional assessments contained in the ICF CoreSets platform.

Despite a generally positive reception of the ICF CoreSets platform, other aspects received mixed feedback and there were many areas of improvement identified by the participants. Much of this feedback was incorporated into revisions of the ICF CoreSets platform. For example, the item about repetitive behaviors, including stimming, was modified so that participants are given the opportunity to report whether they perceive these behaviours as functional, rather than conceptualizing them as exclusively non-functional. This revision aligns with research emphasizing that stimming can be experienced by autistic individuals as a useful self-regulatory behavior observed in neurotypical and atypical individuals (Charlton et al., 2021), despite the potential negative attention it may attract from others (Kapp et al., 2019). From a professional classification point of view, this means that stimming is classified as a potentially impairing symptom of autism in DSM-5 and ICD-11 (American Psychiatric Association, 2013; World Health Organization, 2022), while in ICF nomenclature, it might both be classified as a disabling and enabling body function. The crucial distinction apparently lies in whether stimming is experienced as a burden or serves as a helpful self-regulatory behaviour. Despite being a biopsychosocial framework including almost 1700 functionally descriptive codes allowing individual characterization of an advanced level, even the ICF framework currently embodies a certain disability bias and struggles to fully capture nuances. Thus, the scoring of this specific ICF item operationalizing the ICF code b147 was modified. The ICF CoreSet operationalization and platform in general offers many free-text boxes (after each item, domain, after all items) to add individual perspective and information to compensate for these limitations.

Another example of complying with the perspectives of potential users, particularly neurodivergent informants, were our efforts to modify and add items to better capture masking and energy regulation. Although achieving a comprehensive understanding of how these factors affect functioning in detail remains a matter of research (Lundin Remnélius & Bölte, 2024; Strauß et al., 2018) and therefore may not be fully addressed by a limited number of additional items in the operationalization. Recent research on how autistic individuals experience various rating scales highlights similar challenges in accurately measuring fluctuating energy levels, mood, or roles (Stacey & Cage, 2022). These issues are not easily resolved by common metrics and standardized assessments, thus reinforcing why ICF platform users are encouraged to provide clarifications using the free-text boxes when needed.

Although efforts were made to incorporate participant feedback, some challenges arose in several instances, primarily where different groups held varying perspectives, or the feedback was incongruent with the ICF framework. In some instances, perceptions were partly conflicting; while some individuals expressed a desire for more detailed instructions and more specific items, others favored a streamlined approach. A recurring request was to break down broader items into more detailed items or to include specific examples. However, addressing these requests posed challenges, particularly due to practical and conceptual considerations, such as the length of an already comprehensive assessment or implementing details or examples not specified in the ICF framework, which would result in an inaccurate representation of the original codes.

Various participant groups aiming to represent all potential users were included to provide feedback, which immanently increases the likelihood of receiving diverse opinions due to their unique perspectives. While diversity of experience is a strength of the study, it also presented a challenge in synthesizing the feedback. Consequently, there was a need to strike a balance between accommodating incoming requests, practical feasibility, and, foremost, fidelity to the content of the original ICF codes and ICF framework as such. In implementing changes, we complied with the ICFs’ ethical principles requiring that we prioritize the perspectives of the target group (neurodivergent people). However, it is also of paramount importance to achieve a viable compromize between the perspectives and requests of neurodivergent individuals, relatives, and professionals. The acceptance and utility of the ICF Core Sets platform across relevant arenas, including healthcare, education, and employment, are essential for final use in research and practice. Importantly, the goal of the authors is not to develop from scratch a functional assessment that is optimized for any specific group or context but to make an internationally adopted framework for function usable in real life across groups and arenas.

We received feedback requesting items about personal information such as gender and coping strategies. These particular aspects are encompassed within the domain of personal factors in the ICF. Currently, this domain is uncoded due to its multifaceted nature and the wide variability in its impact on functioning (WHO, 2001). However, in an ongoing project, efforts are being made to code personal factors (Grotkamp et al., 2020) which would enable their inclusion in future assessments. Indeed, while the ICF is comprehensive, it does not encompass all life aspects or essential elements, such as quality of life (Bölte, 2023). Consequently, depending on context and specific needs, other instruments may be valuable complements to the ICF.

Some critiques were raised regarding the ICF-based rating scales’ alignment with neurotypical perspectives on autism and ADHD, particularly highlighting concerns regarding the comparative nature of scoring some functioning dimensions, and the view of certain normative behaviours as strengths. Nonetheless, acknowledging behaviours such as relationship-building, social participation, and health prioritization as strengths is vital, even if it may provoke concerns, as lack of physical activity and social isolation is related to poorer mental health in neurodivergent individuals (Schiltz et al., 2021; Zang, 2019). The notion of making rating scales more neurodivergent-friendly by moving away from comparative norms is certainly a valid argument and demand. However, utilizing some form of standard or norm is also crucial for understanding functioning and for distinguishing differences between sub-groups. This practice can enhance comprehension and support for diverse populations and is a vital component of effective compensation systems. Furthermore, it is inconceivable to disregard the existence of social, economic, or legal norms in society, and they are ultimately essential for societal functionality and cohesion. Still, critique is surely legitimate as ICF classification originates from a disability tradition with a support-oriented focus. Consequently, a more or less subtle “disability perspective” is inherent. One fundamental goal of the ICF is to identify functional disabilities to provide appropriate support and enhance participation (Kostanjsek, 2011). This rationale also explains why we retained the skewed rating scale, allowing more space to describe nuances of difficulties for effective intervention tracking. In practice, capturing difficulties and even minor changes in them is prioritized over highlighting small differences in strengths.

The ICF is a framework taking an international approach across countries, cultures, settings and informants to achieve a global perspective on functioning, taking into account research and keeping organization of services and practice in mind (Leonardi et al., 2022). This comes with the considerable strengths of worldwide consensus, but also implicit limitations when the ICF is applied in a more specified context. Pivotally, the ICF is intentionally a diagnoses free nomenclature (WHO, 2001), which is why adaptations to certain conditions will encounter limitations. This is also true for ICF adaptations to autism and ADHD. On balance, whenever possible within the ICF framework, actions are taken in the development of the ADHD and autism Core Sets to comply with specificities of the conditions and preferences of potential users, particularly general and neurodivergent people. Aside, from conducting the present study, the development of the Core Sets and their validations have seen substantial involvement of stakeholders (Bölte et al., 2024a, 2024b). Preparatory studies of the Core Sets both also highlighted challenges and strengths observed in autism and ADHD (Mahdi et al., 2017, 2018). We also abandoned scoring according to ICF qualifiers, which have a disability format, in favor of a numeric scale capturing a continuum from ability to disability. Finally, free-text boxes enable that any additional relevant content can be added by respondents.

### Strengths and Limitations

The current study includes feedback only from Sweden and the United Kingdom, two high-income Western European countries. The feedback from these two countries had considerable overlap, still, it cannot be considered necessarily representative of a global population, and a different sample might yield other feedback. In saying this, the development of the ICF Core Sets for autism and ADHD was a global endeavour, capturing perspectives across all WHO regions, and has been validated in several countries and regions. As such, there can be some confidence that at least the factors covered by the ICF CoreSets platform are likely globally applicable. Nevertheless, most studies validating ICF Core Sets have been conducted in Europe (Karlsson & Gustafsson, 2022), it is imperative to extend validation efforts to a more diverse array of countries, encompassing both high- and low-income countries. It is worth emphasizing that this is still a rigorous feasibility study, involving two countries and three types of informants in a relatively large sample, with relatively consistent feedback.

It is also worth noting the composition of the focus groups with neurodivergent individuals that included participants with both autism, ADHD, and both. Only the feedback received from the UK was exclusively from autistic adults. Hence, we created similar item-based versions for all neurodivergent groups, despite variations in item composition. This aligns with the overarching principles applicable to these groups, such as the benefits of clarity and structure for both.

A limitation in evaluating the length of the assessment is that the general population and neurodivergent groups responded to different versions. The version received by the general population group contained 170 items, whereas the versions encountered by the neurodivergent group ranged from 156 to 232 items. Consequently, the general population group’s evaluation of the assessment as less cumbersome compared to the neurodivergent group may have been influenced by this discrepancy. Another limitation is the limited sociodemographic profile we were able to develop for the participant sample, owing to missing data and technical issues in the original platform pertaining to this information.

A strength of this study lies in the intricate flowchart, which integrates data from diverse sources. Further, employing multiple-methods design can enhance accuracy and comprehensiveness (Zhang & Creswell, 2013). While validated survey instruments for autistic adults remain scarce (Nicolaidis et al., 2020), our study actively engages with autistic individuals and those with ADHD. With their valuable feedback and additional support from a language custodian, we have made revisions that align with research priorities for the neurodivergent community. These revisions include, for example, clearer item wording and enhanced response options (Nicolaidis et al., 2020). During focus groups, the question “what happens next?” emerged as a valid concern. To address this, we created a summary report as an initial step, providing tangible outcomes for participants. Looking ahead, improving result reporting is essential. Standardizing rating scales would allow for more sophisticated analyses by establishing norms. Additionally, integrating features into reports for cross-participant comparisons and longitudinal analysis would be advantageous. As a second step, we aim to link assessment results to effective, personalized interventions. This involves connecting specific challenges and strengths with evidence-based interventions from research and guidelines. Automatically associating interventions with functional profiles would enhance the practical utility of ICF across domains like education, employment, and healthcare.

## Conclusion

The operationalized Core Sets for autism and ADHD, along with the first version of the ICF CoreSets platform, have been deemed acceptable and user-friendly by neurodivergent individuals, their relatives, and professionals in neurodevelopmental conditions. Leveraging their feedback, which includes suggestions for revising both rating scales and the platform, the user-friendliness is likely to improve. These revisions will be investigated in an upcoming study. Furthermore, usability is expected to improve following the development of norms, allowing for more sophisticated result analyses, as well as linking individual ICF profiles to tailored information on support. The ICF, in general, and ICF Core Sets for autism and ADHD are a work in progress and we expect future developments in this space to further enhance the usefulness of the ICF CoreSets platform.

## Supplementary Information

Below is the link to the electronic supplementary material.

Supplementary file1 (PDF 281 kb)
