# Supplementary material for: Operationalizing the ICF Core Sets for Autism and ADHD: A Multiple-Methods Feasibility Study
Source: J Autism Dev Disord. 2025 Jan 30;56(6):2432–47. doi: 10.1007/s10803-024-06717-4 (PMC13222184; doi:10.1007/s10803-024-06717-4)
Supplement: Supplementary file 1 — Supplementary file1 (PDF 281 kb) [file 10803_2024_6717_MOESM1_ESM.pdf]

Supplementary materials

Table of content

	Pages
**Supplementary table S1**. Overview of all categories and sub-categories	1-2
**Supplementary table S2**. Overview of item changes in the body functions domain	3
**Supplementary table S3**. Overview of item changes in the activities-participation domain	4
**Supplementary table S4.** Overview of item changes in the environmental factors domain	5
**Supplementary table S5**. Overview of design related changes	6

Supplementary table S1. Overview of all categories and sub-categories

Category	Sub-category	Code	Condensed meaning unit	Example quotation	Stated by
Instructions	Clarity	Clarity enhancements	Clarity can be enhanced by changing the order of colours, adding more information, removing information or making the language easier to understand.	"I would have wanted the orange first and the blue in the middle…" (N3), "Do you have to use 'age-appropriate', you could also say something like 'others of the same age'?" (P7), "For my part, it would be good to have a bit more overview of the test layout before I started. Something like a button that says, 'Look here to see what type of questions are coming.'" (R7)	N; R; P
Instructions	Clarity	The instructions were clear	The instructions for the assessment were clear, and the different sections were well described.	"I thought the instructions were good. I understood what I was supposed to do." (N3)	N; P
Instructions	Progress bar	Progress bar	The progress bar was appreciated and helpful for orientation. It was suggested that the progress bar should indicate the remaining items rather than the remaining pages.	"It was like a progress bar at the top, and it was comforting…" (N5)	N; R; P
Items	Appreciated items	Environmental factors	Questions about environmental factors were appreciated.	"I would say, it's a really crucial area… because the environment makes all the difference, and context and surroundings are where difficulties arise." (R8)	N; R; P
Items	Requested additional items	Requested additional topics	Topics that were missing or should be given more focus were, for example, school-setting, mental health, selective eating, masking, perception, energy regulation, gender and coping strategies.	"It doesn't address things that aren't very visible externally, such as masking and the fatigue that can occur during the overload…" (R10)	N; R; P
Items	Length	Too comprehensive	The rating scales were too comprehensive (though some disagreed) and suggestions for shortening it were for example splitting it up into sections, merging similar items or having a dynamic structure.	"It also depends on how sensitive one is to answering these kinds of questions. I think perhaps people who are supposed to answer this might be more okay with it than I am; I just get tired of these things and feel like enough is enough." (P9)<br>"…she [the daughter] thought there were a lot of rephrasing, like asking about the same thing with different sentences and from different angles…it became too cumbersome and repetitive. But as I said, that was her perspective… I didn't react to it because I love forms." (R11), "…if I've answered that I don't go to school, then it should say proceed to question x." (N7)	N; R; P
Items	Appreciated items	Good coverage	The spread of items and the inclusion of strengths were appreciated. Specific items that were mentioned concerned e.g., sleep, perception, and participation.	"You have quite a few questions related to participation, self-determination, and the ability to influence society. I think those aspects are very important because they're not always thoroughly considered in typical assessments." (P10)	N; R; P
Items	Neurotypical perspective	Neurotypical perspective in items	Certain questions were based on a neurotypical perspective, e.g., what was described as weaknesses many perceived as a strength, e.g., stimming.	"…that stimming is perceived as something negative…even though it is natural and healthy." (N9), "Is it really better to be more outgoing and social? I might not want to be super social all the time, but I don't see it as a problem." (N8)	N; R; P
Items	Neurotypical perspective	Norm comparison	The purpose of comparing one person's abilities to others or establishing norms was questioned. It was recommended to place greater emphasis on the individual and the life they aspire to lead.	"If others engage in doing those things, it's quite irrelevant to me…" (N7)	N; R
Items	Length	Forced responses	Forced responses can be frustrating, especially for children with NDCs.	"If the students, for some reason, fail to click on it, they won't proceed to the next page, and it's the kind of thing that can create a lot of frustration." (P5)	P
To respond	Response challenges	Abstract or too general items	Some items were considered abstract or overly broad, requiring clarification by providing concrete examples or specifying the context. Therefore, many items needed clarifications in the comments field.	"There were some questions that were very abstract as well" (P3)	N; R; P
To respond	Response challenges	Compound items	Some items presented two statements simultaneously which was challenging to answer. It was proposed to divide these items into separate components.	"I also thought that certain questions with double assertions were challenging to evaluate. They weren't quite the same thing, which made them difficult to answer." (R9)	N; R; P
To respond	Response challenges	Depends on motivation, context etc	Many items were difficult to answer since functioning depends on motivation, interest, and context (e.g., hormonal factors, energy, other people, etc.)	"It varies depending on where he is. Whether he's with me or someone outside the home, it's like dealing with two completely different people." (R1), "It's one answer when I have energy and feel fully… well and all that, but it's a completely different answer if I'm overstimulated or exhausted." (N7)	N; R; P
To respond	Response challenges	Difficult assessing oneself	Many items presupposed self-awareness. It is difficult to assess oneself, especially when lacking clear comparisons.	"Wow...trustworthy. I don't even know if I'm trustworthy myself." (P8)	N; P

To respond	Response challenges	Difficult words	Some wording was hard to understand due to ambiguity or complex vocabulary.	"I don't even understand what that means, and I've read up on it since. I've realized there's a psychological term for it, but regular people don't say like that." [about "shared attention"] (N7)	N; R; P
To respond	Response challenges	Item preamble disappears	The introductory text for certain items became obscured when scrolling, which was frustrating.	"In several places, there's a beginning of a question at the top, and then it's just 'friends', 'family', or whatever it might be." (P5)	N; P
To respond	Response challenges	What time frame	Some items were difficult to understand regarding if it asks about the current situation or how it was when they were young, e.g. items about school.	"It was formulated in such a way that it was difficult to know whether one should answer based on how it was when they were in school or if it only applied to those currently attending school. So it was a bit difficult to answer, you know." (N12)	N; R
To respond	Item scaling	Clear scale	The response scale was comprehensible and functioned effectively.	"I thought it worked well. I think it felt clear." (P11)	P
To respond	Item scaling	Colouring of scale	Issues with the use of colours in the response scale; some thought the colours were judgmental, that contrast was poor or preferred a grey scale.	"Do we really need these colours at all?" (N1)	N; R; P
To respond	Item scaling	Comment field	The comment field after each item was appreciated.	"And it was good that you could comment on and elaborate on the answer." (N12)	N; R; P
To respond	Item scaling	Comment field - too short	The comment field after each item was too limited.	"I wrote a lot of comments, but the characters didn't always suffice for me. However, I understand that you don't want essays for every little question." (R5)	N; R
To respond	Item scaling	Omit buttons - unclear	It was difficult to understand when to use the omit buttons, and their wording was unclear.	"What's the difference between 'Unknown' and 'Not applicable'?" (N8)	N; R; P
To respond	Item scaling	Sub-optimal design	The response scale had a sub-optimal design (asymmetry, too many steps, too rigid, etc) and received suggestions on enhancement.	"There are so many steps; how can one determine if it's a one, two, or three? Perhaps there are too many steps in it." (R10), "I was a bit bothered that this 'typical function' wasn't centered…" (N12), "But I would like to be able to have some form of weighting because it's the case that certain areas have more, or rather some can have extremely strong significance, and others are sort of half-important." (R11)	N; R; P
To respond	Item scaling	Use of colours	Using colours in the instructions and the response scale was appreciated.	"So, I think it's pedagogical to use different colours. And it also becomes clear what falls within the normal range, which gives you an idea of how the scale is graded." (R7)	N; R; P
To respond	Item scaling	Worded scales	The wording under the response scale ("Atypical-Typical-High function") was confusing or found marginalising or judgmental.	"Why do they actually call it 'Atypical functioning' and 'High functioning'? For me, it almost feels a bit marginalizing…" (N1)	N; R; P
To respond	Item scaling	Words in the response scale	The use of words in the response scale was appreciated as it added context to the numbers.	"I really want the words because they're the only thing that gives context to what this means, because I really don't like assessment scales, you know." (N5)	N
Usability	Enhanced accessibility	Improved accessibility	Accessibility of the platform could be increased, e.g., by improving contrast, adding text-to-speech functionality, and adding more visual aids.	"I think about people who don't have complete colour vision. Isn't it impossible for many people to distinguish between red and green?" (R5)	N; R
Usability	Feedback	Result summary	It would be appreciated to receive a summary after completing all the items.	"Something I missed a little was perhaps that you could go back to your answers so you could reflect on it…" (N5)<br>"From our perspective, both my child and I have wondered what happens next. The child says that they don't remember their answers very well, so there's some curiosity about what happens afterward. Will we get to see any results?" (R5)	N; R; P
Usability	The platform	User-friendly	The design of the platform was visually pleasing, easy to navigate and overall user-friendly.	"The text is clear, and… I think so too, especially with the fonts and such. So I thought it was good." (N8)<br>"It feels like it's designed in a way that makes it very intuitive." (P8)	N; R; P
Usability	The platform	Areas of application	The platform would be helpful to use in school- and/or work-settings and would have a good potential for collaboration and as a basis for communication.	"It would be incredibly helpful to have this type of knowledge and communication support." (R11)	R
Usability	The platform	Useful and relevant	Overall, the platform was thought of being relevant and a good initiative.	"Honestly, I think it's incredibly relevant, and I'm really happy that you're working on this. I'm very excited to see what the results will be." (R11)	N; R; P

Note. *N = Neurodivergent; R = Relative; P = Professional*

Supplementary table S2. Overview of item changes in the body functions domain

**Subdomain**	**Item changes**
Behavioural style	b125b: Rephrasing<br>b125d-e: Rephrasing
Energy and drive	b130a-d: Rewording<br>b130e: New item
Memory	b144b: Rephrasing<br>b144d: Rephrasing<br>b144e: New item
Motor functions and coordination	b147: Rephrasing<br>b760: Rephrasing
Unusual movements	b765a-b: Rephrasing<br>*Subdomain renamed*
Perception	b156a-e: Rewording
Problem solving	b117: Rewording<br>*Subdomain renamed*
Sensory processing	*Changes to response scale*[a]
Language and body language	b167d: Rewording
Speech	b330a-d: Rewording
Temperament personality	b126a-h: Rewording
Thinking	b160a: Rephrasing<br>b160c: Rephrasing<br>b160d: Clarified examples<br>b164a-b: Clarified examples<br>b164c-d: Rephrasing
Attention	b140d: Removed

Note. *Rephrasing* entails more comprehensive changes to the item, while *Rewording* entails simple changes such as editing single words. *Clarified examples* entails changes or additions to examples given. [a] The response scale that originally went from "-5 to 0" was changed to go from "-5 to +5".

Supplementary table S3. Overview of item changes in the activities-participation domain

Subdomain	Item changes	Subdomain	Item changes
Applying knowledge	d175a-b: Rewording	Self-care	d550d: Rewording
Social interaction	d710a: Removed<br>d710d-e: Rewording<br>d710j: Rephrasing<br>d720: Rephrasing<br>d720b-c: New Item<br>d730: Rewording and clarified examples	Community life	d910: New item<br>d910a-d: Removed (replaced by d910)<br>d920a: Rewording<br>d920b: Rephrasing<br>d930c-d: Removed (replaced by d920b)<br>d930: Clarified examples
Work	d845: New item<br>d845a-c: Removed (replaced by d845)<br>d845d: Removed	Learning	d130: Rewording and clarified examples<br>d132b: Rewording<br>d132c: Rewording<br>d155a-b: Rewording and clarified examples
Empowerment	d940a: Rewording	Use of senses and focus	d160a-b: Rephrasing<br>d161: Rewording
Economics	d860a: Rephrasing and clarified examples<br>d870a: Clarified examples	Conversations	d350a-d350e[a]: Rephrasing<br>d350f-g: New item
Preschool, elementary school and high school	*Addendum to header and preamble*	Social relations	d740b: Rephrasing and clarified examples<br>d760a: Rephrasing (split into d760a-b)<br>d760b: New item
Giving information	d330b: Rewording (changed to d330)	Playful activities gaming	d880a-d: Rewording
Household chores	d640d: Removed<br>d650: New item<br>d650a-b: Removed (replaced by d650)<br>d650c: Removed	Performing tasks	d210a: Clarified examples<br>d210b: Rewording and clarified examples<br>d230a: Rewording and clarified examples<br>d220: Rewording
Health behaviours	d570a-b: Rewording<br>d570e: Rewording<br>d570g-h: Rewording<br>d570i: Clarified examples	College, university, and other education	No changes to items<br>*Preamble added*
Use of transport	d470: Rephrasing<br>d475a-b: Rephrasing<br>d475c: Rewording	Receiving information	d310a: Rephrasing (split into d310a-b)<br>d310b: New item<br>d315c: Rewording

Note. *Rephrasing* entails more comprehensive changes to the item, while *Rewording* entails simple changes such as editing single words. *Clarified examples* entails changes or additions to examples given. [a] The items received new codes.

Supplementary table S4. Overview of item changes in the environmental factors domain

**Subdomain**	**Item changes**
Support and relationships	e350: New item<br>*Preamble added*
Assistive products and technology	No changes to items
Communication services	e535a. Rephrased<br>e535d: Rephrased
Diet and supplements	No changes to items<br>*Preamble changed*
Light and sound conditions	No changes to items
Medicines	No changes to items
Media	e560a-c: Rephrasing<br>e560d: Rewording
The attitudes of others	e440: Rephrasing<br>e460: Removed<br>e460a-b: New items (replaced e460)<br>*Preamble added*
Community services	e550: Rewording<br>e575: Clarified examples<br>*Preamble added*
School and education	No changes to items

Note. *Rephrasing* entails more comprehensive changes to the item, while *Rewording* entails simple changes such as editing single words. *Clarified examples* entails changes or additions to examples given.

Supplementary table S5. Overview of design related changes

Area	Issue	Description	Action
Response scale	Colours in the response scale	Participants did not appreciate the colours, especially red/orange, which were perceived as negative and judgmental.	Changed the red/orange to purple.
Response scale	Response scale labels	The labels "Atypical function; Typical function; High function" were confusing and perceived as evaluative.	Removed "Atypical function; Typical function; High function" from the rating scale.
Response Scale	Additional response buttons	The two omit buttons "Unknown; Not applicable" caused confusion.	Renamed "Unknown" to "I don't know" and "Not applicable" to "Not relevant".
Instructions	The order in which the colours are described	Confusion because the order of the colours was different in the instructions then the rating scale.	Changed the order in the instructions to match the order in the rating scale; purple, blue, green.
Comment field	Space in the comment field was too limited	Insufficient space in the comment field after each question.	Two extra comment boxes were added after each domain, with unrestricted space. Previously, there was only one such box at the end of the entire Core Set.
Summary report	The response report was unclear.	Difficult to read the response report and requests to condense the response report.	A new response report was added, providing a simple summary of the highest and lowest rated items per domain.
Accessibility	Suggestion to make the design more accessible	The colours of the response scale could be difficult to perceive for colour blind people and the text hard to read for people with dyslexia.	An accessibility tool was implemented with functions such as changing contrast, screen reader, reading mask etc.
